# Estimating the impacts of nonoptimal temperatures on mortality: A study in British Columbia, Canada, 2001–2021

**DOI:** 10.1097/EE9.0000000000000303

**Published:** 2024-03-14

**Authors:** Rudra K. Shrestha, Ioana Sevcenco, Priscila Casari, Henry Ngo, Anders Erickson, Martin Lavoie, Deena Hinshaw, Bonnie Henry, Xibiao Ye

**Affiliations:** a Office of the Provincial Health Officer, Ministry of Health, Government of British Columbia, Victoria, BC, Canada; b School of Environment and Sustainability, Royal Roads University, Victoria, British Columbia, Canada; c Data Science and Health Research Cluster, Faculty of Medicine, University of British Columbia, Vancouver, British Columbia, Canada; d Health Protection Branch, Population and Public Health Division, Ministry of Health, Government of British Columbia, Victoria, British Columbia, Canada; e School of Population and Public Health, University of British Columbia, Vancouver, British Columbia, Canada; f School of Health Information Science, University of Victoria, Victoria, British Columbia, Canada

**Keywords:** Nonoptimal temperature, Heat dome, British Columbia, Canada, Attributable deaths

## Abstract

**Background:**

Studies show that more than 5.1 million deaths annually are attributed to nonoptimal temperatures, including extreme cold and extreme heat. However, those studies mostly report average estimates across large geographical areas. The health risks attributed to nonoptimal temperatures in British Columbia (BC) are reported incompletely or limit the study area to urban centers. In this study, we aim to estimate the attributable deaths linked to nonoptimal temperatures in all five regional health authorities (RHAs) of BC from 2001 to 2021.

**Methods:**

We applied the widely used distributed lag nonlinear modeling approach to estimate temperature–mortality association in the RHAs of BC, using daily all-cause deaths and 1 × 1 km gridded daily mean temperature. We evaluated the model by comparing the model-estimated attributable number of deaths during the 2021 heat dome to the number of heat-related deaths confirmed by the British Columbia Coroners Service.

**Results:**

Overall, between 2001 and 2021, we estimate that 7.17% (95% empirical confidence interval = 3.15, 10.32) of deaths in BC were attributed to nonoptimal temperatures, the majority of which are attributed to cold. On average, the mortality rates attributable to moderate cold, moderate heat, extreme cold, and extreme heat were 47.04 (95% confidence interval [CI] = 45.83, 48.26), 0.94 (95% CI = 0.81, 1.08), 2.88 (95% CI = 2.05, 3.71), and 3.10 (95% CI = 1.79, 4.4) per 100,000 population per year, respectively.

**Conclusions:**

Our results show significant spatial variability in deaths attributable to nonoptimal temperatures across BC. We find that the effect of extreme temperatures is significantly less compared to milder nonoptimal temperatures between 2001 and 2021. However, the increased contribution of extreme heat cannot be ruled out in the near future.

**What this study adds:**

This is the first study to report temperature-related attributable deaths in all five regional health authorities (RHAs) of British Columbia (BC). The findings are consistent with those reported in global studies, which show that moderate cold is the primary contributor to nonoptimal temperature-related attributable deaths. The findings, however, also indicate a significant amount of variability across the different RHAs. The application of fine-resolution gridded population data, which consider the average distribution of people throughout a typical day, and climate data to minimize population exposure measurement errors is another valuable contribution to epidemiological research.

## Introduction

Frequency and magnitude of extreme weather events, including heat and cold waves, are projected to intensify across Canada,^1^ leading to climate-induced attributable deaths and hospitalizations. The impacts have already been observed in the province of British Columbia (BC), where it is estimated that more than 600 deaths were linked to the 2021 heat dome, a period of extreme heat in the summer of 2021.^2–4^ The heat dome also caused a sudden increase in heat-related illness and emergency department visits in Portland and Seattle in the United States.^5,6^ Furthermore, the Government of BC issued an extreme cold alert in February 2023 and opened warming centers across the province to mitigate the impacts.^7^

**Figure 1.**
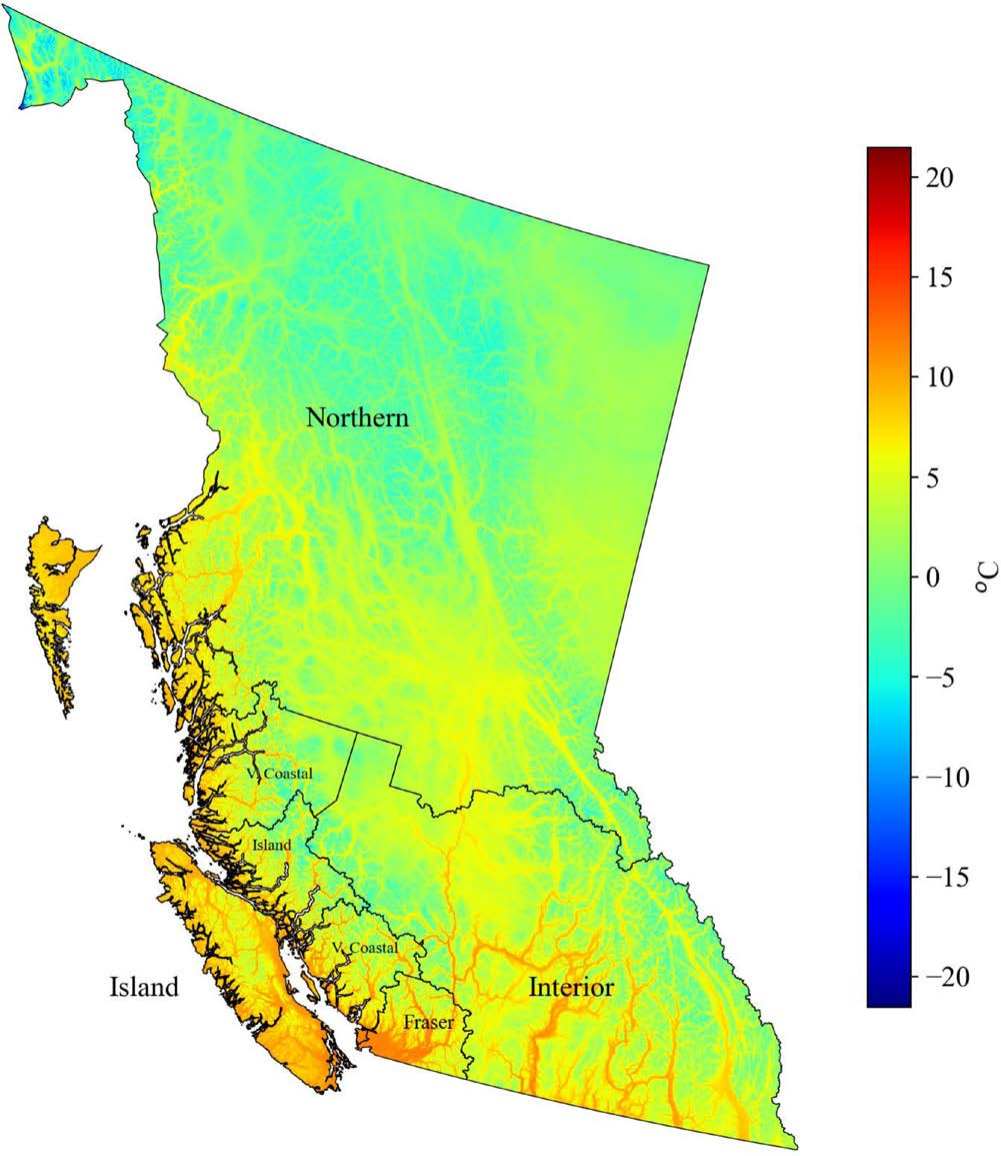
Gridded (1 × 1 km) daily mean temperature averaged between 2001 and 2021, also showing the boundaries of the regional health authorities in BC.

A number of global scale studies have reported that approximately 10% of attributable deaths are due to nonoptimal temperatures,^8^ roughly equivalent to 5.1 million deaths per year, and the majority of them are attributed to cold.^9,10^ In Europe, 223,793 average annual attributable deaths were reported between 2000 and 2019.^10^ However, the burden varies substantially between countries, as the temperature–mortality association depends on climatic conditions, geographic location, and population characteristics.^10–12^ The association is further complicated by socioeconomic and infrastructural factors.^13,14^ Such complexity can be apparent across BC due to its diverse geography and climate (Figure 1), which warrants a more detailed regional and community scale study.

Few such studies have been conducted in BC,^15–17^ and most of them are limited in scope as they either concentrate only on the effects of extreme heat on population health outcomes using station-based climatic data or limit the study area to a particular urban center. Hence, a comprehensive study at the regional scale is necessary to quantify the impact of climate change on attributable deaths across BC. In this study, using high-resolution gridded climate and population data, we present deaths attributable to nonoptimal temperatures over 21 years (2001–2021) across all five regional health authorities (RHAs) in BC, representing nearly 13% of the population of Canada.

## Methods

### Study design and data sources

The study includes all five RHAs in BC: Fraser, Interior, Island, Northern, and Vancouver Coastal. We collected climate, population, and mortality data for complete calendar years from 2001 to 2021 for each RHA, as described below.

We obtained 1 × 1 km gridded daily maximum and minimum surface temperatures from the Daily Surface Weather and Climatological Summaries (Daymet).^18^ Grid-scale daily mean temperatures were calculated following Spangler et al.^19^ Likewise, 1 × 1 km gridded 24-hour averaged annual population data were obtained from LandScan,^20^ which considers the diurnal movements of the population and collective travel habits. Population-weighted average temperature (hereafter temperature) is calculated following Tol.^21^

We determined the daily time series of all-cause mortality from the BC Vital Statistics Agency’s deaths registry.^22^ Death geography was based on the decedent’s residential address at the time of death from BC Healthcare Client Roster.^23^ Only BC residents were included. We combined dissemination area-level deprivation indices obtained from the Institut national de santé publique du Québec (INSPQ)^24^ to determine the proportion of the population living in high deprivation census dissemination areas by RHA across all the study years. We used the BC Chronic Disease Registry^25^ to determine the percentage of people living in a region with at least one chronic condition. This study used heat-related deaths confirmed by the British Columbia Coroners Service (BCCS) during the 2021 heat dome to evaluate the modeling results. The BCCS multidisciplinary death review panel investigated different aspects of deaths, including physical and circumstantial evidence of deceased individuals, to identify the cause of death.^2^ This study is conducted as part of a population health research program approved by the University of British Columbia Research Ethics Board (Ethics REB # H22-01818).

### Statistical analysis

We used a two-stage modeling approach^5^ that has been widely used to model exposure–lag–response association.^26–28^ In the first stage, we applied a distributed lag nonlinear model with a quasi-Poisson distribution in each RHA to estimate the temperature–mortality association. The regression model includes a natural cubic B-spline of time with eight degrees of freedom per year, controlling seasonal and long-term trends and day of the week using indicator variables. A quadratic B-spline with three internal knots placed at the 10th, 75th, and 90th percentile of temperature distribution was used for the exposure–response association. A natural cubic B-spline with an intercept and three equally spaced internal knots in the log scale with lags up to 21 days were used for the lag–response association. The RHA-specific bidimensional exposure–lag–response associations were reduced to overall cumulative exposure–response. This one-dimensional association reduces the number of modeling parameters but maintains the complexity of the relationship and is reported as relative risk (RR) with respect to a reference level.

In the second stage, we fitted the RHA-specific overall cumulative exposure–response association using a multivariate meta-analytic model,^29^ including RHA-specific meta-predictors, average temperature, percent of the population with at least one chronic disease, and percent of the population residing in a high social deprivation area, which reduce a substantial amount of heterogeneity and reported using *I*^2^ statistic (Table S1; http://links.lww.com/EE/A264). We used a best linear unbiased prediction^30^ to derive an improved estimate of exposure–response association.^8,28,29^

We quantified the attributable fraction and number of deaths^31^ using RHA-specific best linear unbiased prediction and temperature distribution. For each RHA, we estimated the deaths attributable to four different temperature ranges: moderate heat, extreme heat, moderate cold, and extreme cold. These temperature ranges serve as cutoff points for evaluating temperature-related mortality risks. Moderate heat was defined as a temperature ranging between the minimum mortality temperature (MMT),^8^ a reference temperature at which mortality risk is lowest, and the 97.5th percentile of the temperature distribution, while extreme heat is defined as a temperature that exceeds the 97.5th percentile of the temperature distribution. Similarly, moderate cold is a temperature ranging between MMT and the 2.5th percentile of the temperature distribution, and extreme cold represents a temperature below the 2.5th percentile of the temperature distribution. The empirical confidence intervals (eCIs) of the temperature–mortality association were calculated using Monte Carlo simulations. The “dlnm”^32^ and “mvmeta”^29^ packages were used in R software (version 4.0.5, R Foundation for Statistical Computing, Vienna, Austria) for the analyses.

## Results

We analyzed 703,638 all-cause deaths in BC between 2001 and 2021. Fraser and Northern RHAs recorded the highest and lowest number of all-cause deaths, respectively (Table 1). Records show that Northern RHA is the coldest region in BC, with an average daily temperature of 5.5 °C, which is approximately 5 °C colder than other RHAs. On the other hand, based on the mean temperatures, Fraser RHA is the hottest region in BC.

**Table 1.** Descriptive statistics by regional health authority (RHA), 2001–2021

Regional health authority	Total deaths	Population-weighted temperature (°C)^a^	Population with at least one chronic disease (%)^b^	Population with high social deprivation (%)^b^
Fraser	220,855	11.4 (0.1 to 22.6)	36.1 (27.1 to 39.8)	31.6 (29.2 to 32.9)
Interior	147,620	9.3 (−8.5 to 25.3)	38.8 (28.2 to 43.6)	47.4 (43.8 to 48.6)
Island	150,569	10.6 (1.5 to 20.3)	40.1 (30.2 to 44.7)	56.1 (52.2 to 62.4)
Northern	39,974	5.5 (−15.0 to 20.1)	34.1 (23.0 to 40.0)	49.5 (41.9 to 53.7)
Vancouver Coastal	144,620	11.1 (0.6 to 21.7)	32.7 (24.7 to 36.2)	45.4 (42.6 to 48.5)

a Mean (2.5th percentile to 97.5th percentile).

b Mean (range).

**Figure 2.**
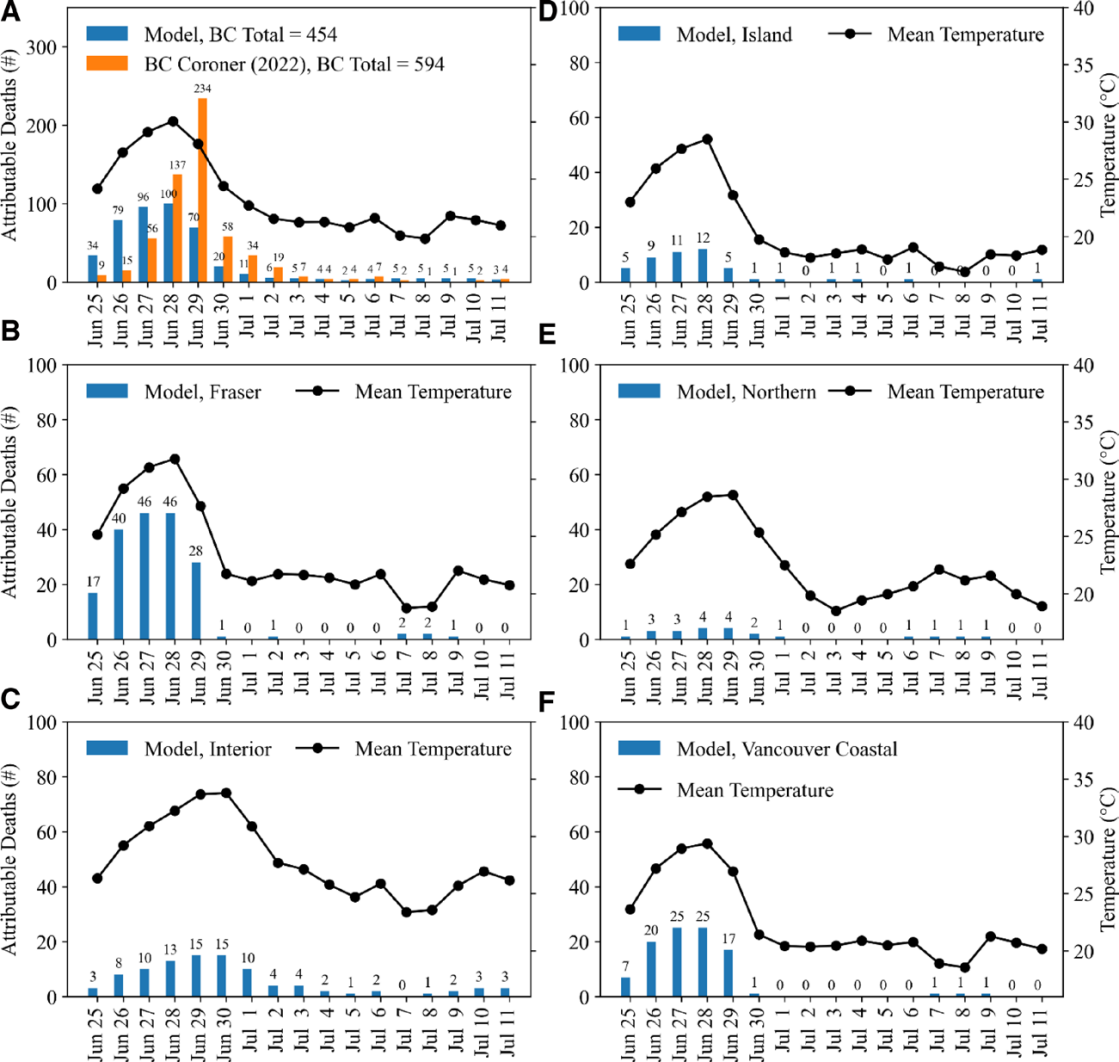
A comparison of model-estimated heat-related deaths in BC and deaths confirmed by the BC Coroner’s Service (A), and model-estimated heat-related deaths in each RHA (B–F), between 25 June and 11 July 2021. The corresponding daily mean temperature is shown on the secondary *y*-axis.

We used the 2021 heat dome event to evaluate the model results (Figure 2A) by comparing the heat-attributable deaths estimated from our model against the number of heat-related deaths confirmed by the BCCS^33^ for the reference period between 25 June and 11 July. Our model estimated a total of 454 heat-related attributable deaths during the heat dome as opposed to the 594 heat-related deaths reported by the BCCS. We also did a similar calculation for each RHA (Figure 2B–F), but the BCCS data are not available for comparison. In general, the model picked up the early surge of heat-related mortality during the onset of the heatwave, peaking at the time when the maximum daily mean temperatures were recorded. However, the observed peak mortality in BC, as shown in the BCCS report, lagged by 1 day (Figure 2A).

Following the model evaluation, we used the model for retrospective analysis. From 2001 to 2021, we estimated that 7.17% (95% eCI = 3.15, 10.32) of deaths in BC were attributed to nonoptimal temperatures. This includes 6.24% (95% eCI = 2.44, 9.59) due to moderate cold, 0.13% (95% eCI = −0.11, 0.36) due to moderate heat, 0.38% (95% eCI = 0.22, 0.52) due to extreme cold, and 0.42% (95% eCI = 0.31, 0.51) due to extreme heat. On average, the mortality rates attributable to moderate cold, moderate heat, extreme cold, and extreme heat were 47.04 (95% confidence interval [CI] = 45.83, 48.26), 0.94 (95% CI = 0.81, 1.08), 2.88 (95% CI = 2.05, 3.71), and 3.10 (95% CI = 1.79, 4.4) per 100,000 population per year, respectively. Results showed that the highest overall attributable fraction of deaths for all nonoptimal temperatures combined (9.19%) is estimated in Island RHA as opposed to 3.31%, the lowest fraction, in Northern RHA. In BC and all RHAs, we found that the relative contribution of moderate cold to total mortality attributable burden is significantly higher as compared to the contributions of extreme cold, moderate heat, and extreme heat, which are less than 1% (Table 2).

**Table 2.** Attributable fraction of deaths by regional health authority (RHA), with aggregated values for BC

Regional health authority/province	MMT (°C)	Overall (%)	Moderate cold (%)	Moderate heat (%)	Extreme cold (%)	Extreme heat (%)
British Columbia	20.2	7.17 (3.15, 10.32)	6.24 (2.44, 9.59)	0.13 (−0.11, 0.36)	0.38 (0.22, 0.52)	0.42 (0.31, 0.51)
Fraser	20.9	7.89 (−1.1, 15.12)	7.09 (−1.37, 14.16)	0.06 (0.01, 0.1)	0.19 (−0.12, 0.52)	0.55 (0.36, 0.7)
Interior	20.2	4.58 (−1.04, 9.41)	3.73 (−1.84, 8.63)	0.16 (−0.2, 0.51)	0.37 (0.13, 0.59)	0.32 (0.13, 0.48)
Island	16.6	9.19 (2.47, 15.54)	8.18 (1.5, 13.98)	0.21 (−0.3, 0.7)	0.51 (0.21, 0.8)	0.3 (0.06, 0.53)
Northern	8.8	3.31 (−0.66, 6.7)	2.06 (−3.83, 6.54)	0.34 (−3.43, 3.6)	0.58 (0.2, 0.94)	0.33 (−0.19, 0.75)
Vancouver Coastal	20.2	7.65 (−3.3, 16.64)	6.65 (−3.38, 15.58)	0.05 (−0.01, 0.1)	0.5 (0.1, 0.81)	0.46 (0.22, 0.66)

Values in parentheses represent 95% confidence intervals (CIs).

The overall cumulative temperature–mortality association curves show minimal risk due to moderate heat, characterized by a lower RR. However, the risk increases dramatically when the ambient temperature rises above the extreme heat threshold. The contribution of the 2021 heat dome is significant as excluding the year 2021 from the analysis leads to flattening the temperature–mortality association beyond the threshold. Note that the risk associated with extreme heat only accounts for a small fraction of days, as indicated in the annual temperature distribution. The cold-related risk generally increases slowly and nonlinearly with a decrease in temperature below MMT (Figure S1; http://links.lww.com/EE/A264). However, the evolution pattern of the cold-related risk is not consistent across all RHAs. In the Interior and Northern RHAs, the risk increases with decreases in temperature, as expected, whereas in Fraser, Island, and Vancouver Coastal RHAs, the risk shows the opposite trend, decreasing on extremely cold days, although the uncertainty is high.

**Figure 3.**
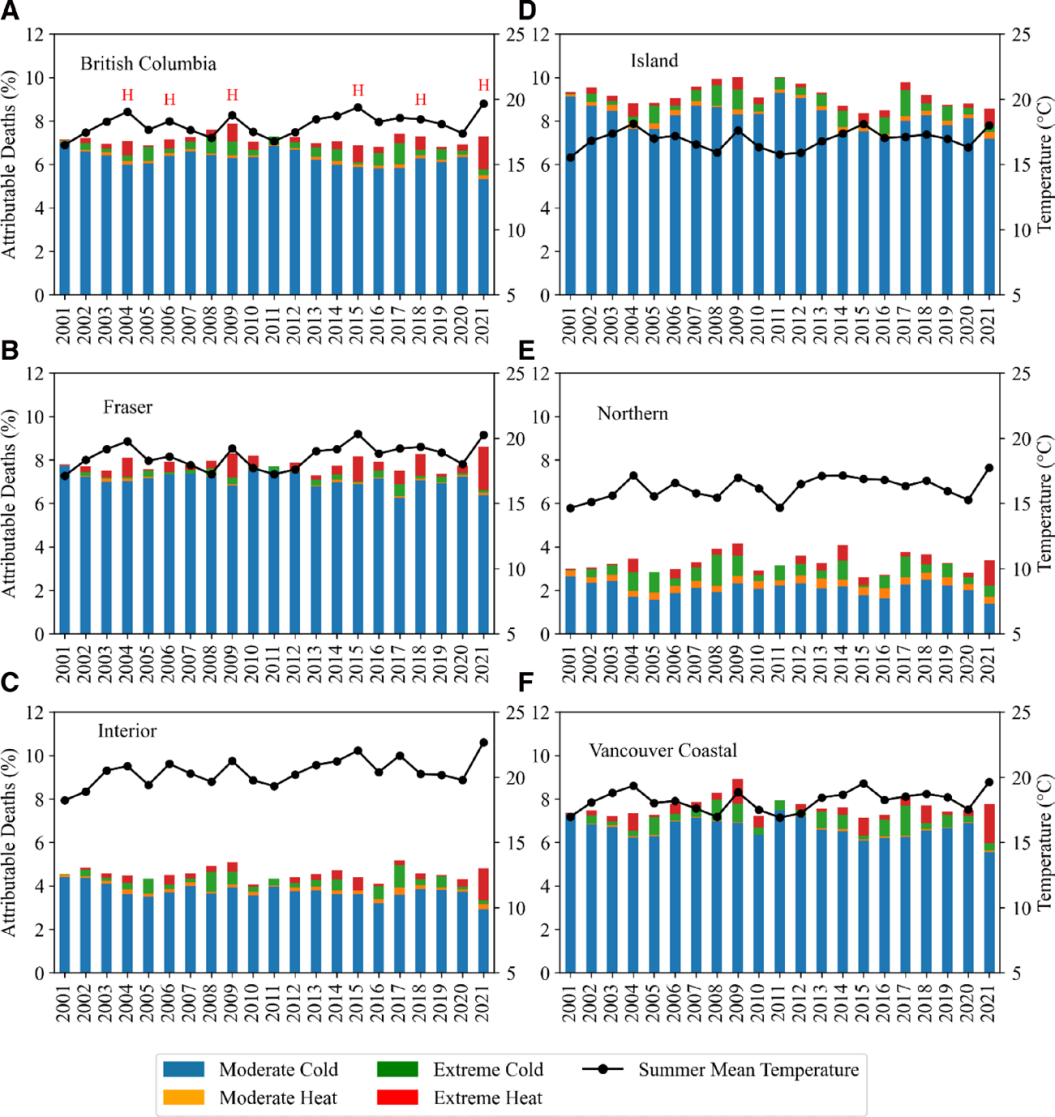
Annual attributable deaths in BC (A) and all five RHAs (B–F), along with the corresponding summer mean temperature (solid black line) from 2001 to 2021. The letter "H" in (A) represents a heat wave year.

**Figure 4.**
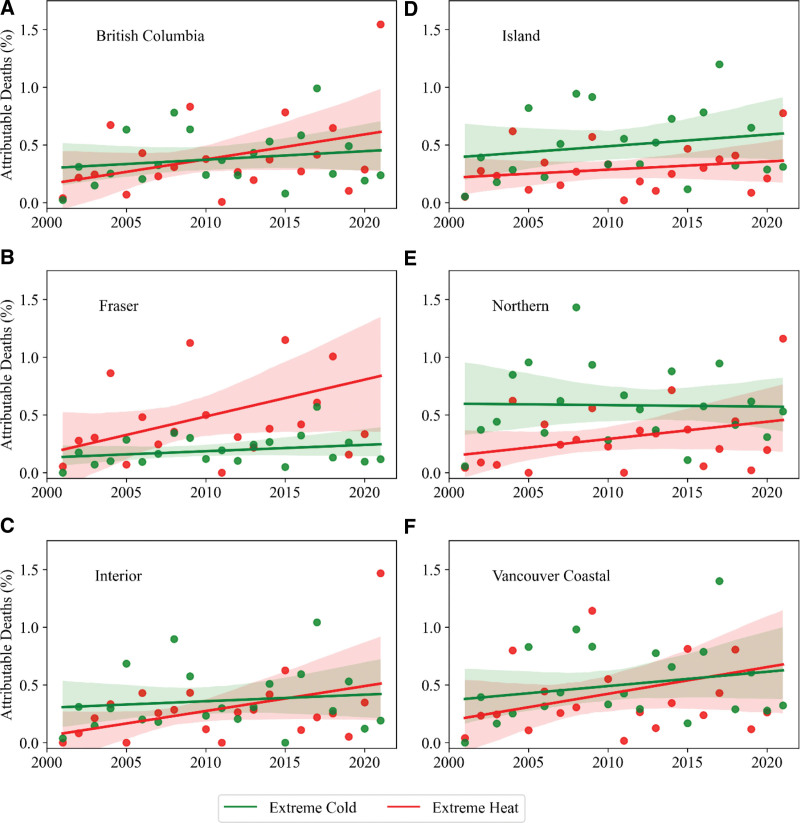
A trend analysis of the attributable fraction of deaths in BC (A) and all five RHAs (B–F) due to extreme heat and extreme cold. Dots represent the annual attributable fraction of deaths, and solid lines represent the corresponding trend lines. The shading areas denote 95% CI.

Figure 3 shows the total fractions of deaths attributable to nonoptimal temperatures in BC and all five RHAs, as well as the contribution of extreme cold, moderate cold, extreme heat, and moderate heat. Overall, within all RHAs, the total fractions of annual deaths attributable to nonoptimal temperatures do not vary substantially from year to year. However, the contributions of extreme cold, moderate cold, extreme heat, and moderate heat show variations over the 21-year period. A trend analysis (Figure 4) shows that the attributable fraction of deaths due to extreme temperatures is increasing in all five RHAs, with more pronounced effects of extreme heat in Fraser. However, the moderate temperatures show minimal change or a decreasing trend (Figure S2; http://links.lww.com/EE/A264). The contribution of extreme heat is significant during heat wave years, indicated by the letter “H” in Figure 3A, and consistent with summer mean temperatures (solid black line). As shown in Figure 3, moderate cold is the dominant cause of temperature-related death in BC. Additionally, the total fractions of attributable deaths due to nonoptimal temperatures are significantly higher in Fraser, Vancouver Coastal, and Island RHAs compared to Interior and Northern RHAs.

## Discussion

In this study, we used a distributed lag nonlinear model to investigate deaths attributable to nonoptimal temperatures in BC. The model results were evaluated against the heat-related deaths confirmed by the BCCS during the 2021 heat dome in BC. Overall, the model-estimated heat-related deaths in BC between 25 June and 11 July 2021 were approximately 25% lower than the heat-related deaths confirmed by the BCCS during the same period. However, we argue that the discrepancy does not necessarily mean the model underestimated heat-related deaths, as the observation data may have limitations. The BCCS follows a protocol to review each individual death by considering physical and circumstantial evidence, possibly contributing to the discrepancies. For example, for the death investigation, BCCS used weather data from the nearest station, which may be too far and/or at a different altitude from the place of injury, potentially influencing the conclusions.^2^ Furthermore, total counts of heat-related deaths in BC during the 2021 heat dome have been reported inconsistently.^2,3^

We also found differences between the daily number of heat-attributable deaths estimated by our model and the daily count of heat-related deaths reported in the BCCS report. In particular, our model estimates the initial surge of deaths during the onset of the heat wave, peaking on 28 June, consistent with the time when the majority of BC experienced the hottest temperatures. However, the BCCS investigation shows a slightly delayed death surge, which peaks on 29 June. This 1-day lag in the timing of peak mortality is potentially attributed to the delay in case reporting, as 98% of the deaths occurred at home and 56% of the decedents were living alone.^2^ Additionally, the temporal discrepancy may also be attributed to the complexity of the model, which uses various degrees of nonlinear basis, cross-basis functions, and a complex lag structure to estimate attributable deaths.

We estimated that 7.17% of all-cause deaths in BC between 2001 and 2021 were attributed to nonoptimal temperature, the majority of which are attributed to cold (6.62%), contributing 92% to the temperature-related attributable deaths. In comparison, heat-related deaths (0.55%) were minimal in this time period and only contributed 8% to the total of nonoptimal temperature attributable deaths. It should be noted that the impact of extreme heat on mortality may increase in the future unless appropriate adaptation measures are implemented, as extreme heat is anticipated to become more common with the impacts of climate change.^34^ Our estimate of heat-related mortality is similar to the one reported by Gasparrini et al,^8^ who used the same approach and estimated 0.54% excess deaths due to nonoptimal heat in 20 Canadian cities from 1986 to 2009 and also consistent with 0.44% in 297 counties of the United States.^35^

Cold-related deaths in BC were estimated to be 6.62%, which is greater than the 4.46% across Canada reported by Gasparrini et al.^8^ Several factors may have contributed to this inconsistency. First, we use high-resolution gridded climate data with algorithms that have been validated in complex terrain, in contrast to Gasparrini et al, who used station-based climate data and included only two cities from BC in their analysis. Second, we applied population-weighted mean temperature, which reflects the temperature experienced by the population and could significantly affect the temperature–mortality association in the geographically heterogeneous region,^19,36^ instead of mean daily station-based land surface temperature as an exposure index. Because weather stations are generally placed far from densely populated areas, which may enhance exposure measurement errors. Third, our temporal coverage from 2001 to 2021 is another key difference. Changing weather patterns may have affected the results, especially since extreme weather events are becoming more frequent due to climate change. Finally, we used a unique set of meta-predictors that capture socioeconomic status and underlying health conditions of the populations.

A study conducted in Switzerland,^28^ which arguably has socioeconomic conditions and mountain-dominated geography similar to BC, estimated 8.91% of cold-related deaths, higher than our finding of 6.62%, using a comparable statistical modeling framework and utilizing a population-weighted temperature driven by gridded climate data. Such discrepancies between studies suggest that further research is necessary at the community level representing different geographic, demographic, socioeconomic, and infrastructural factors.

Our results show substantial spatial variability in overall attributable fractions of deaths (Table 2) across BC. Island, Vancouver Coastal, and Fraser RHAs had a higher fraction of deaths, whereas Northern and Interior RHAs showed a lower fraction. Several factors account for the variability in attributable deaths across the RHAs. First, temperature–mortality association depends on socioeconomic, climatic, and demographic factors.^12,13^ BC comprises four ecoregions characterized by unique microclimates^15^ and a wide range of socioeconomic and demographic structures.^37^ For example, among the five RHAs in BC, Island has a large percentage of the population experiencing both high social deprivation and living with at least one chronic disease (Table 1), which could explain the higher fraction of attributable deaths. However, the temperature–mortality relationship is very complex and cannot be interpreted the same across all RHAs.

Another reason that may account for the variability in attributable deaths across the RHAs is the spatial variability of climate. An accurate representation of climate variability in the model allows us to estimate the precise temperature thresholds and MMTs at which mortality risk rises or falls within each RHA. Previous studies suggested that widely spaced weather stations induce bias and may not accurately estimate population exposure metrics.^38^ The bias is prominent in complex geography, where the exposure of interest (e.g., temperature) varies significantly with space.^39^ Hence, the application of detailed exposure metrics along with ambient population data in our analysis may have contributed to the observed variability across the RHAs.

Our results suggest a significant temporal and spatial variability in the annual fraction of attributable deaths due to extreme temperatures across RHAs (Figure 4). While Fraser, Vancouver Coastal, and Interior RHAs show increasing trends in the deaths attributable to extreme heat, consistent with an increasing trend of extreme heat events in BC, we found less pronounced or even decreasing trends due to extreme cold. However, Northern and Island RHAs show more steady trends for extreme heat and cold-related annual attributable fraction of deaths between 2001 and 2021. Furthermore, our results indicate broadly stable and decreasing trends in attributable deaths due to moderate heat and moderate cold, respectively. The extreme heat that occurred in 2004, 2006, 2009, 2015, 2018, and 2021 stand out, and they are consistent with the spikes in summer mean temperatures across BC (Figure 3), and the impacts were reflected in the annual attributable deaths. In particular, significant contributions of extreme heat to the nonoptimal temperature-related attributable deaths were observed across all the RHAs.

Consistent with previous research, RR associated with nonoptimal cold temperatures increases nonlinearly when ambient temperature drops below MMT. The risk generally decreases at extreme temperatures across all RHAs, which could either be an artifact due to the “rebound” of the spline curve or a real “protective effect” since people stay indoors on these extreme cold days and are not exposed.^40^ Similar effects were simulated across several cities in the United States, South Korea, Canada, and Sweden (Figure S1 in ^8^). In contrast, RR increases drastically when the ambient temperature rises above the extreme heat thresholds, which is consistent with a multicountry observational study (Figure S1 in ^8^), which found an exponential increase in risk at high ambient temperatures across several cities in Spain, China, Italy, United Kingdom, and the United States.

Our study has some limitations. We have yet to adjust for the impacts of harmful pollutants (e.g., ozone, particulate matter) and humidity on population health outcome due to the lack of data availability. Furthermore, in this first phase of the project, we analyzed the temperature–mortality association on a broader geographical level in BC. Using lessons from this study, we are conducting further research focusing on community-level exposure–response associations, results will be published in the near future.

In summary, our findings highlight the effects of nonoptimal temperatures on attributable deaths across different RHAs in BC. The observed variability emphasizes the significance of taking into consideration local factors, including climatic, socioeconomic, and demographic characteristics to understand temperature–mortality associations. Moreover, it is also important to understand the multifaceted nature of the temperature–mortality association, including the multidimensional effects of a heat wave.^41,42^ A heat wave increases the direct risk of mortality and morbidity of people with underlying health conditions as they are more sensitive to extreme heat. The heat wave also increases the risk indirectly by creating a favorable environment for surface-level ozone formation. Hence, heat waves act as a “mortality accelerator,” and, such events will be more intense and frequent, as projected by climate models,^43^ warranting further research focusing on the complex interactions among extreme temperatures, population vulnerability, and air quality.

## Conflicts of interest statement

The authors declare that they have no conflicts of interest with regard to the content of this report.

## Supplementary Material


