# Supplementary material for: Estimating the impacts of nonoptimal temperatures on mortality: A study in British Columbia, Canada, 2001–2021
Source: Environ Epidemiol. 2024 Mar 14;8(2):e303. doi: 10.1097/EE9.0000000000000303 (PMC11008660; doi:10.1097/EE9.0000000000000303)
Supplement: Supplementary file 1 [file ee9-8-e303-s001.docx]

**eAppendix**

**Estimating the Impacts of Non-Optimal Temperatures on Mortality: A Study in British Columbia, Canada, 2001-2021**

Rudra K. Shrestha, Ioana Sevcenco, Priscila Casari, Henry Ngo, Anders Erickson, Martin Lavoie, Deena Hinshaw, Bonnie Henry, Xibiao Ye

**Contents:**

1. Figure S1: Overall cumulative temperature – mortality association curve in five regional health authorities of British Columbia (BC).
2. Figure S2: A trend analysis of the attributable fraction of deaths due to moderate heat and moderate cold. Dots represent the annual attributable fraction of deaths, and solid lines represent the corresponding trend lines. The shading areas denote 95% CI.
3. Table S1. I^2^ statistic (%) in different multivariate random-effects meta-regression models.





Figure S1: Overall cumulative temperature – mortality association curve in all five regional health authorities of British Columbia (BC), with 95% confidence interval, shaded grey. The distribution of temperature for each RHA is also shown. The solid grey line represents minimum mortality temperatures (MMT), and the dashed grey lines on the left and right sides of the MMT represent 2.5^th^ and 97.5^th^ percentiles, respectively. RR = relative risk.


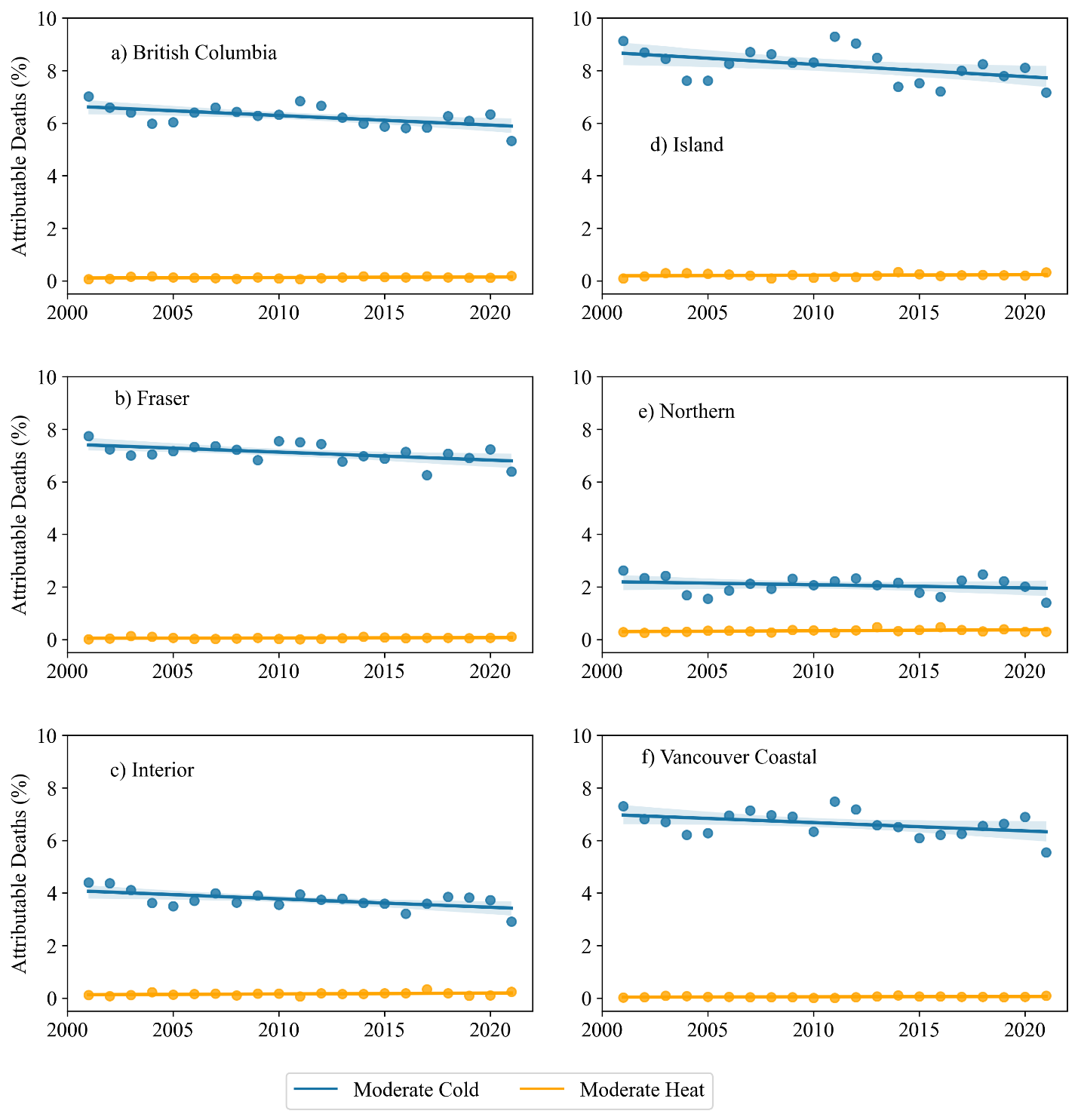


Figure S2: A trend analysis of the attributable fraction of deaths due to moderate heat and moderate cold. Dots represent the annual attributable fraction of deaths, and solid lines represent the corresponding trend lines. The shading areas denote 95% CI.

Table S1. I^2^ statistic (%) in different multivariate random-effects meta-regression models.

| Model | Predictor | I^2^ |
| --- | --- | --- |
| Base model | - | 54% |
| Single predictor | Percent of the population with at least one chronic condition | 51% |
|  | Mean temperature | 49% |
|  | Social deprivation index | 0 |
| Full model | Percent of the population with at least one chronic condition |  |
|  | Mean temperature | 0 |
|  | Social deprivation index |  |
